# Influence of Bariatric Surgery on Oral Microbiota: A Systematic Review

**DOI:** 10.1055/s-0042-1753471

**Published:** 2022-09-08

**Authors:** Hafiz Adawi, Aparna Aggarwal, Saurabh Jain, Maha A. Othman, Ahlam A. A. Othman, Rawan A. Zakri, Somayah A. M. Namazi, Sara A.Y. Sori, Lamees H. A. Abuzawah, Zainab M. Madkhali

**Affiliations:** 1 Department of Prosthetic Dental Sciences, College of Dentistry, Jazan University, Jazan, Saudi Arabia; 2 Private Practice, Vitaldent Dental Clinic, Faridabad, Haryana, India; 3 Experimental Oral Pathology, Barts and The London School of Medicine and Dentistry, Queen Mary University of London, London, United Kingdom; 4 Department of Fixed Prosthodontics, Faculty of Dentistry, Sana'a University, Sana'a, Yemen; 5 Hayati Care Clinic, Sabya, Jazan, Saudi Arabia; 6 College of Dentistry, Jazan University, Jazan, Saudi Arabia

**Keywords:** bariatric surgery, gingival crevicular fluid, microbiota, saliva, periodontal disease, mouth

## Abstract

The study aims to systematically review the available literature to evaluate the changes in oral microbiota in patients after bariatric surgery (BS) and correlates these alterations in microorganisms with common oral manifestations. Relevant Electronic databases were systematically searched for indexed English literature. The Preferred Reporting Items for Systematic Reviews and Meta-analyses (PRISMA) guidelines were followed for framework designing, application, and reporting of the current systematic review. The focused PICO question was: “Is there any change in oral microbiota (O) of patients (P) who underwent BS (I) when compared with non-BS groups (C)?' Seven articles were selected for qualitative synthesis. On application of the National Institutes of Health (NIH) quality assessment tool, six studies were found to be of fair quality and one was of good quality. All the seven included studies evaluated the effect of BS on oral microbiota in humans. The outcomes of this review suggest that considerable changes take place in oral microbiota after BS which can be correlated with common oral manifestations. These changes are mainly due to the indirect effect of BS and may vary with the individuals. Due to variations in the included studies, it is difficult to proclaim any persistent pattern of oral microbiota found after BS.

## Introduction

Obesity is defined as an abnormal or excessive fat accumulation that presents a risk to health. 1 As per the World Health Organization (WHO), there is an increase in obese people (body mass index [BMI]>30kg/m ^2^ ) in both developed and developing countries. When compared with the year 2000, there is a 1.5 times increase in obesity among adults (18 years and older) and more than two times increase in children (5–19 years) in 2016. 2 Thirty-nine million children under the age of 5 years were overweight or obese in 2020. 3 Bariatric surgery (BS) is one of the effective treatment modalities to manage morbidly obese patients and their related comorbidities in the long term. 4 Different types of weight reduction surgeries are documented, but the most commonly performed surgeries are Roux-en-Y gastric bypass (RYGB) and sleeve gastrectomy (SG). 5 Rapid loss of excessive weight due to BS improves the quality of life and decreases the mortality rate in these morbidly obese patients by reducing the related comorbidities like type-2 diabetes mellitus (DM), diabetes complications, hyperlipidemia, steatohepatitis, hypertension, cardiovascular disorders, respiratory disorders, varicose veins, and others. 6 7

Various systemic manifestations associated with post-BS procedures include gastric ulcerations, gastroesophageal reflux, vomiting, diarrhea, nutritional deficiencies, and others. 8 9 These systemic changes, directly or indirectly, result in oral manifestations like dental caries, 10 dental erosion, 11 dental wear, 12 periodontitis, 12 13 mucosal alterations, 14 sialometric changes, 12 15 sialochemical changes, 13 16 and taste alterations. 17 18

Gastrointestinal (GI) microbiota has been shown to affect the gut–brain axis by their involvement in inflammatory and metabolic responses. 19 20 Studies have reported that there is a change in GI microbiota in patients undergoing BS. 21 22 This change in microbiota, along with anatomic rearrangement and alteration in GI hormone levels, leads to surgery-mediated weight loss. 23 24 The oral cavity, being an integral part of the alimentary tract, is also reported to have altered microbiota in patients undergoing BS. 13 15 16 25 26 27 28 These oral microbial changes can alter the oral environment which along with other factors (changes in salivary flow 12 15 and salivary composition 13 16 ) can increase the risk of oral diseases. 29

As per our knowledge, to date, there is no systematic review that assesses the change in oral microbiota after BS. The findings are potentially vital as these may guide dentists in preventing damage to the oral cavity and can help medical specialists in relating them with other systematic changes commonly seen in patients after BS. The study aims to systematically review the available literature to evaluate the changes in oral microbiota in patients after BS and to correlate these alterations in microorganisms with common oral manifestations. The hypothesis framed is that there is no change in oral microbiota in patients after BS.

## Methods

Guidelines given by the Preferred Reporting Items for Systematic Reviews and Meta-analyses (PRISMA) were used in framework designing, application, and reporting of the current systematic review. 30 The protocol was registered with the International Prospective Register of Systematic reviews and was assigned the following identification code: PROSPERO CRD42021267677.

### Selection Criteria

Inclusion and exclusion criteria are listed in Table 1 .

**Table 1:** Inclusion and exclusion criteria

Inclusion criteria	Exclusion criteria
Literature in the English language	Literature in a language other than English
Human clinical studies	Animal and cadaver studies
Studies only comparing changes in oral microbiota before and after BS, irrespective of sex and age	Letter to the editor, opinion based commentaries, dissertations, review papers, abstract presentations, and incomplete trials
Studies comparing oral microbiota of patients who underwent BS with non BS group, irrespective of sex and age	Studies reporting oral microbiota post BS, without comparing it with oral microbiota before BS or in non BS groups
	Studies only comparing changes in salivary flow, chemical composition of saliva and oral health after BS
	Studies reporting changes in GI microbiota only

Abbreviations: BS, bariatric surgery; GI, gastrointestinal.

### Exposure and Outcome

The exposure of interest for the current study was any form of BS, irrespective of the method (type of surgery) or time (duration after the surgery). The outcome was the change in oral microbiota after BS. The focused PICO/PECO (participant, intervention/exposure, comparison, and outcome) question was: “Is there any change in oral microbiota (O) of patients (P) who underwent BS (I) when compared with non-BS groups (C)?”

### Search Strategy, Study Selection, and Data Extraction

Electronic databases (PubMed/Medline, PubMed Central, Web of Science, and Cochrane library) were systematically searched by two independent reviewers (S.J. and A.A.) for articles published from 1987 to January 30, 2022. Different groups of Medical Subject Heading (MeSH) terms and supplementary non-MeSH terms were used. Details of search strings and Boolean operators are mentioned in Table 2 . Duplicate articles were removed, and there was no discrepancy in the two lists of articles. H.A. and S.J. analyzed the titles and abstracts of all the articles based on predefined inclusion and exclusion criteria. If relevant information could not be obtained, the full text of the article was reviewed. A Manual search was conducted by searching Google, *clinicaltrials.gov* , and references of shortlisted articles to identify relevant articles. The selected articles were cross-checked by A.A. Full texts of shortlisted articles were reviewed by S.J. and A.A., and based on the predetermined exclusion and inclusion criteria, appropriate studies were selected. Any disagreements or differences in opinions were discussed with another reviewer (H.A.), and a consensus was reached.

**Table 2:** Electronic databases and research strategies

Database	Combination of terms used for search	Number of titles
PubMed/Medline	("bariatrics"[MeSH Terms] OR "bariatric a "[Title/Abstract] OR "Bariatric Surgery"[MeSH Terms] OR "gastroplasty"[MeSH Terms] OR "Jejunoileal Bypass"[MeSH Terms] OR "Gastric Bypass"[MeSH Terms] OR "Sleeve gastrectomy"[Title/Abstract] OR "Weight Loss Surgery"[Title/Abstract] OR "duodenal-jejunal bypass"[Title/Abstract] OR "gastrojejunostomy"[Title/Abstract] OR "DJB"[Title/Abstract] OR "RYGB"[Title/Abstract]) AND ("saliva"[MeSH Terms] OR "saliva a "[Title/Abstract] OR "Oral"[Title/Abstract] OR "mouth"[MeSH Terms] OR "periodontium"[MeSH Terms] OR "periodontal ligament"[MeSH Terms] OR "gingiva"[MeSH Terms] OR "Gingival Crevicular Fluid"[MeSH Terms] OR "GCF"[Title/Abstract]) AND ("microbiota"[MeSH Terms] OR "Microbiome"[Title/Abstract] OR "Microflora"[Title/Abstract] OR "Microbial"[Title/Abstract] OR "microbiology"[MeSH Terms] OR "microbio a "[Title/Abstract] OR "mycobiome"[MeSH Terms] OR "bacteria"[MeSH Terms] OR "fungi"[MeSH Terms])	44
PubMed Central	(((("microbiota"[MeSH] OR "Microbiome"[tiab] OR "Microflora"[tiab] OR "Microbial"[tiab] OR "microbiology"[MeSH] OR "microbio a "[tiab] OR "mycobiome"[MeSH] OR "bacteria"[MeSH] OR "fungi"[MeSH]))) AND (("saliva"[MeSH] OR "saliva a "[tiab] OR "Oral"[tiab] OR "mouth"[MeSH] OR "periodontium"[MeSH] OR "periodontal ligament"[MeSH] OR "gingiva"[MeSH] OR "Gingival Crevicular Fluid"[MeSH] OR "GCF"[tiab]))) AND ("bariatrics"[MeSH] OR "bariatric a "[tiab] OR "Bariatric Surgery"[MeSH] OR "gastroplasty"[MeSH] OR "Jejunoileal Bypass"[MeSH] OR "Gastric Bypass"[MeSH] OR "Sleeve gastrectomy"[tiab] OR "Weight Loss Surgery"[tiab] OR "duodenal-jejunal bypass"[tiab] OR "gastrojejunostomy"[tiab] OR "DJB"[tiab] OR "RYGB"[tiab])	1,923
Web of Science	#1 (TS=(bariatrics OR bariatric a OR "Bariatric Surgery" OR gastroplasty OR "Jejunoileal Bypass" OR "Gastric Bypass" OR "Sleeve gastrectomy" OR "Weight Loss Surgery" OR "duodenal-jejunal bypass" OR "gastrojejunostomy" OR DJB OR RYGB) ) AND **LANGUAGE:** (English) Indexes=SCI-EXPANDED, SSCI, A&HCI, CPCI-S, CPCI-SSH, ESCI, CCR-EXPANDED, IC Timespan=All years #2 (TS=(saliva OR saliva a OR Oral OR mouth OR periodontium OR "periodontal ligament" OR gingiva OR "Gingival Crevicular Fluid" OR GCF) ) AND **LANGUAGE:** (English), Timespan=All years #3 (TS=(microbiota OR Microbiome OR Microflora OR Microbial OR microbiology OR microbio a OR mycobiome OR bacteria OR fungi) ) AND **LANGUAGE:** (English), Timespan=All years #3 AND #2 AND #1 Indexes=SCI-EXPANDED, SSCI, A&HCI, CPCI-S, CPCI-SSH, ESCI, CCR-EXPANDED, IC Timespan=All years	52
Cochrane Library	#1MeSH descriptor: [Bariatrics] explode all trees #2bariatric a #3MeSH descriptor: [Bariatric Surgery] explode all trees #4MeSH descriptor: [Gastroplasty] explode all trees #5MeSH descriptor: [Jejunoileal Bypass] explode all trees #6MeSH descriptor: [Gastric Bypass] explode all trees #7"Sleeve gastrectomy" #8"Weight Loss Surgery" #9"duodenal-jejunal bypass" #10"gastrojejunostomy" #11DJB #12RYGB #13MeSH descriptor: [Saliva] explode all trees #14saliva a #15Oral #16MeSH descriptor: [Mouth] explode all trees #17MeSH descriptor: [Periodontium] explode all trees #18MeSH descriptor: [Periodontal Ligament] explode all trees #19MeSH descriptor: [Gingiva] explode all trees #20MeSH descriptor: [Gingival Crevicular Fluid] explode all trees #21GCF #22MeSH descriptor: [Microbiota] explode all trees #23Microbiome #24Microflora #25Microbial #26MeSH descriptor: [Microbiology] explode all trees #27microbio a #28MeSH descriptor: [Mycobiome] explode all trees #29MeSH descriptor: [Bacteria] explode all trees #30MeSH descriptor: [Fungi] explode all trees #31#1 OR #2 OR #3 OR #4 OR #5 OR #6 OR #7 OR #8 OR #9 OR #10 OR #11 OR #12 #32#13 OR #14 OR #15 OR #16 OR #17 OR #18 OR #19 OR #20 OR #21 #33#22 OR #23 OR #24 OR #25 OR #26 OR #27 OR #28 OR #29 OR #30 #34#31 AND #32 AND #33	11

a Truncation was used to broaden the sea.

Relevant data, extracted from the final articles, were tabulated in a self-designed table ( Table 3 ). The data extracted were as follows: first author's name, year of publication, the country where the study was conducted, study type ( *in vitro* or *in vivo* ), objects, the objective of the study, sample size (number of patients), gender, mean age, mean BMI of participants (before and after surgery), presence of comorbidities, oral diagnosis/findings, type of BS, microbiota investigation technique, location of specimen collection, time of specimen collection, change in levels of the microbiome, reported oral changes after BS, correlation of altered species with oral and general manifestations, and authors suggestions/conclusions.

**Table 3:** Main characteristics of the studies included

**Study (year)**	**Place of the study**	**Study type and objects**	**Objective of the study (related to oral microbiome)**	** Sample size ( *n* ), gender, and age **	** Mean BMI of participants (kg/m ^2^ ) **	**Systemic comorbidities**	**Oral diagnosis/findings**	**Type of BS**	**Microbiota investigation technique**	**Location of specimen collection**	**Time of specimen collection**
Sales-Peres et al (2015) 13	Brazil	*In vivo* (humans)	To evaluate the influence of BS on periodontal disease and on the quantity of periodonto-pathogenic bacteria in morbid obese patients	*n* =50 (42 F, 8M) Mean age: 38.90±10.13 years	Before BS: 49.69±9.97 6 months after BS: 36.16±.05 12 months after BS: 32.26±5.78	–	Periodontitis	RYGB	qPCR specifically targeting 4 specific periodontal pathogens ( *P. gingivalis,* *Treponema denticola, Tannerella forsythia* , and *P. itermedia* ).	GCF	•Before BS •6 Months after BS 12 Months after BS
Hashizume et al (2015) 15	Brazil	*In vivo* (humans)	To evaluate the salivary conditions of morbidly obese patients before and after BS	*n* =27 (26 F, 1M) Average age: 45±8 years	Before BS: 51.72 (±4.52) 6 months after BS: 38.02 (±5.46)	High BP ( *n* =20), DM ( *n* =14)	Dental prosthesis wearers ( *n* =10), dental caries ( *n* =11)	RYGB	selective media to specifically culture and quantitate 2 dental caries associated bacterial groups (mutans Streptococci and *Lactobacilus sp* .) and the fungal yeast species *Candida albicans*	Stimulated saliva	•Before BS 6 Months after BS
Stefura et al (2021, 2020) 28 32	Poland	*In vivo* (humans)	To analyze using the microbiota of patients with morbid obesity undergoing BS	*n* =46 Group 1: BS (EWL>50%) *n* =19 (13 F, 6M); mean age: 40.44±8.62 years Group 2: BS (EWL<50%) *n* =11 (9F, 2M); mean age: 46.72 ±14.46 years Group 3: non-BS *n* =16 (7F, 9M); mean age: 42.47±8.59 years	Median maximal BMI Group 1: Before BS: 49.2 (43.4–55.5) After BS: 41.1 (39.3–49.2) Group 2: Before BS: 50.3 (45.5–51.5) After BS: 48.4 (45–49.7) Group 3: Non-BS: 51.62 (47.5–54.37)	Type-2 DM, diabetes complications, hyperlipidemia, steatohepatitis, hypertension, cardiovascular disorders, respiratory disorders, varicose veins	–	laparoscopic SG	NextGen (Illumina) Sequencing (targeting V3 and V4 regions of 16sRNA gene)	Oral swabs	Groups 1 and 2: •Before BS •6 months after BS Group 3: •Along with groups 1 and 2 (before BS)
Džunková et al (2020) 26	Czech Republic	*In vivo* (humans)	To describe the salivary microbiome changes during body weight loss on an individual-specific level, and to elucidate the effect of BS on the salivary microbiome	*n* =35 (17 F, 18 M) Average age: 48±9 years	Before BS: 44.99±7.73 3 months after BS: 38.95±7.04 12 months after BS: 35.9±5.6	–	–	Four: SG (N=3), RYGB (N=5), Omega loop gastric bypass (N=7), laparoscopic gastric plication (N=20)	NextGen (Illumina) Sequencing targeting V3 and V4 regions of 16sRNA gene	Unstimulated whole mouth saliva	•Before BS •1 day after BS 3 months after BS
Balogh et al (2020) 27	Hungary	*In vivo* (humans)	To investigate the effect of weight loss on the crevicular microbiota following BS	*n* =57 Non-BS normal control: *n* =22 (13 F, 9M) Mean age: 33.9 years (18–53) Non BS Obese controls: *n* =18 (13 F, 5M) Mean age: 44.1 years (19–58) BS group: *n* =17 (7F, 10 M) Mean age: 39.4 years (21–54 years)	Non-BS normal control: 23.3 (SD=2.16) Non BS obese controls: 44.5 (SD=10.79) BS group: Before BS: 46 (SD=7.03) After BS: 31.5 (SD=8.3)	No comorbidities	No periodontitis	Not mentioned	Identification by matrix-assisted laser desorption ionization–time of flight mass spectrometry (MALDI-TOF MS) and MALDI Biotyper	GCF	•Non BS group BS group: •Before BS 11.3 months (average) after BS
Pataro et al (2016) 25	Brazil	*In vivo* (humans)	To compare the frequency of oral periodonto-pathogens and *Helicobacter pylori* in the mouths of obese individuals with or without periodontitis, subjected to BS	*n* =154 (121 F, 33 M) Mean age: 37.58±11.36 years BS group: *n* =79 Non-BS group: *n* =75	BS group: (26.89±4.48 and 26.53±4.23) Non-BS group: (41.65±4.7 and 39.89±7.08)	–	Periodontitis ( *n* =75)	RYGB	qPCR specifically targeting 5 specific periodontal pathogens ( *P. gingivalis, Aggregatibacter actinomycetemcomitans, Parvimonas micra, T. denticola, T. forsythia, C. rectus* ) and 1 stomach pathogen ( *H. pylori* )	Unstimulated whole saliva and scrapings from the tongue dorsum	•Non BS group At least 24 months after BS (39.37±15.80)
Shillitoe et al (2012) 16	The United States	*In vivo* (humans)	To examine differences in oral microbes in obese patients with and without type-2 DM, and to determine whether it is feasible to measure changes after BS	*n* =29 (22 F, 7M) Mean age: 41 years (range: 23–55)	Before BS: 48 (37–97) 2 week after BS: approximately 43 12 week after BS: approximately 38	Type 2 DM ( *n* =13)	No periodontitis	RYGB	qPCR specifically targeting 3 gastrointestinal bacterial groups ( *Firmicute spp* ., *Bacteroidites spp* ., and *Bifidobacteria spp.* ) and three specific species ( *Bacteroides thetaiotaomicron, P. gingivalis,* *Methanobrevibacter smithii),* with only *P. ginigivalis* , specifically associated with the oral cavity because this study also assessed stool specimens	Stimulated saliva	•Before BS 2 Weeks after BS
**Study (year)**	**Change in levels of microbiome**					**Reported oral changes after BS**		**Correlation of altered species with oral and general manifestations**		**Authors suggestions/ conclusions**	
Sales-Peres et al (2015) 13	A. Frequency of bacteria in GCF of individuals B. With regard to relative quantity of bacteria 1. Changes after 6 months of surgery: a. Statistically significant increase: *P. gingivalis* and *T. forsythia* b. Statistically non-significant increase: *T. denticola* and *P. intermedia* c. Statistically significant or non-significant decrease: none 2. Changes after 12 months of surgery (in comparison to 6 months) a. Statistically significant decrease: *P. gingivalis* b. Statistically nonsignificant decrease: *T. forsythia* , *T. denticola* & *P. intermedia* c. Statistically significant or nonsignificant increase: none 3. Changes after 12 months of surgery (in comparison to Pre BS) a. Increase: *P. gingivalis* , *T. forsythia* b. Decrease: *P. intermedia* , *T. denticola*					• Increase in the severity of periodontal disease (increase in pocket depth, increased loss of clinical attachment, increase in bleeding index)		• *P. gingivalis* , *T. forsythia* , *T. denticola* and *P. intermedia* : +ve correlation with periodontal disease • *P. gingivalis* : +ve correlation with cardiovascular disease		• Increase in the quantity of periodonto-pathogenic bacteria after BS • Increase in the severity of periodontal disease after BS • Increase in *P. gingivalis* after BS, which can increase the risk of cardiovascular disease	
Hashizume et al (2015) 15	Changes after 6 months of surgery: a. Statistically significant increase: Mutans streptococci b. Statistically nonsignificant increase: *C. albicans* c. Statistically nonsignificant decrease: *Lactobacillus spp* .					–		• *S. mutans* : +ve correlation with dental caries • *C. albicans* : +ve correlation with oral candidiasis, ‒ve or no correlation with caries and +ve correlation with systemic diseases like DM, Sjögren's syndrome, and in immunosuppression		• Increase in salivary levels of mutans streptococci • More focus on oral health of BS patients, both before and after BS, to prevent or to minimize oral and systemic manifestations, related to changes in oral microbiota	
Stefura et al (2021, 2020) 28 32	Most abundant microbiota: In group 1 after BS: proteobacteria, burkholderiaceae, betaproteobacteria, *Lautropia* , burkholderiales, *Capnocytophaga* , *Saccharofermentans* , neisseriales, neisseriaceae, *Facklamia* , *Acidaminococcaceae* , *Acidaminococcus* , *Morococcus* In group 2 after BS: micrococcaceae, micrococcales, *Rothia* , actinobacteria, bacillales, *Gemella* , *Siccibacter* In group 3: *Trabusiella* , *Colidextribacter*					–		• Phyla proteobacteria: +ve correlation with gastritis • Phyla bacteroidetes: +ve correlation with periodontal disease. • Phylum actinobacteria: +ve correlation with dental caries		• Percentage of expected weight loss after BS determines the nature of oral microbiota after BS and these are independent of demographic and perioperative characteristics of the patients	
Džunková et al (2020) 26	1. Changes after 1 day of BS: intraindividual level revealed heterogeneity of changes in salivary microbiome composition a.Increase by more than 100% in more than 50% of patients: firmicutes; *Veillonella atypica* b. Increase by 5–100% in more than 50% of patients: firmicutes; *Granulicatella adiacens* c. Decrease by more than 100% in more than 50% of patients: proteobacteria; *Haemophilus parainfluenzae* , firmicutes; *Gemella sp* ., *Granulicatella elegans* , *Porphyromonas endodontalis* , *Bergeyella sp* . d.Decrease by 5–100% in more than 50% of patients: None 2. Changes after 3 months of BS: The patient-specific changes did not show uniform direction of microbiome changes a Increase by more than 100% in more than 50% of patients: *Veillonella atypica* , *Megasphaera micronuciformis* , and *Prevotella salivae* b Increase by 5–100% in more than 50% of patients: None c. Decrease by more than 100% in more than 50% of patients: *Granulicatella elegans* , *Porphyromonas pasteri* , *Gemella sp* ., *Prevotella nanceiensis* c. Decrease by 5–100% in more than 50% of patients: *Streptococcus oralis* 3. Changes after 12 months of BS: No significant increase or decrease in in species at end of 3 months and 12 months					–		• *Veillonella atypica* : early colonizers in oral biofilm formation along with streptococcus • *Megasphaera micronuciformis* : −ve correlation with caries • *Prevotella salivae* : +ve correlation with periodontal disease		• Heterogeneous change in salivary microbiome • Multiple individual specific factors influence the salivary microbiome more than the reduction in BMI	
Balogh et al (2020) 27	BS group when compared before and after BS A. Number of positive samples: 1. increase in number of positive samples: a.Significant increase: *Candida* b.Nonsignificant increase: *Prevotella* , *Staphylococcus* , *Haemophilus* , *Eikenella* , *Fusobacterium* , *Veillonella* 2. Decrease in number of positive samples: a.i)Significant decrease: *Neisseria* b.ii) Nonsignificant decrease: *Actinomyces* , *Granulicatella* 3. No change in number of positive samples: *Rothia* , *Streptococcus* B. Mean germ count: 1. Increase in mean germ count: a Significant increase: *Candida* , *Streptococcus* b Nonsignificant increase: Prevotella, Neisseria, Haemophilus, Staphylococcus, Eikenella 2. Decrease in mean germ count: a Significant decrease: none b Nonsignificant decrease: *Actinomyces, Fusobacterium, Granulicatella, Veilonella* 3. No change in mean germ count: *Rothia* a Significant increase in germ count of *Streptococcus* after surgery b -Non-albicans *Candida* species ( *C. dubliniensis* , *C. kefyr* , and *C. lusitaniae* ) emerged after surgery, both in terms of the proportion of patients and a significant germ count surge					• No periodontitis (no signs of inflammation, no attachment loss greater than 3mm)		• *C. albicans* : +ve correlation with oral candidiasis, −ve or no correlation with caries and +ve correlation with systemic diseases like DM, Sjögren's syndrome, and in immunosuppression • *Prevotella* : +ve correlation with periodontitis, acute necrotizing ulcerative gingivitis • Nonalbicans *Candida* species ( *C. dubliniensis* , *C. kefyr* , and *C. lusitaniae* ): +ve correlation with immunosuppression • *Neisseria* : −ve correlation with periodontitis		• Nonsignificant increase in germ count after BS • Unlikely to develop periodontitis after BS if patients have healthy periodontium, good oral hygiene maintenance, and no predisposing factors preoperatively • Vital to examine oral cavity and treat any periodontal disease before BS	
Pataro et al (2016) 25	1. In nonperiodontitis patients. Frequency of bacteria in BS group when compared to non BS group a Increase in frequency by more than 100%: *P. gingivalis* , *T. denticola* , red complex (simultaneous presence of *P. gingivalis* , *T. denticola* and *T. forsythia* ) b Increase in frequency between 5–100%: *T. forsythia* , C. rectus c. Decrease in frequency by more than 100%:None d Decrease in frequency between 5–100%: *H. pylori* , *A. actinomycetemcomitans* , *P. micra* 2. In Periodontitis patients. Frequency of bacteria in BS group when compared to non BS group a Increase in frequency by more than 100%: *P. gingivalis* , *T. denticola* , red complex b Increase in frequency between 5–100%: *A. actinomycetemcomitans* , *T. forsythia* c. Decrease in frequency by more than 100%:None d. Decrease in frequency between 5–100%: *H. pylori* , *P. micra* , *C. rectus*					• Loss of periodontal tonus • Increase in bleeding on probing		• Red complex (simultaneous presence of *P. gingivalis* , *T. denticola* and *T. forsythia* ): +ve correlation with periodontal disease • Actinomycetemcomitans: +ve correlation with periodontal disease • *H. pylori* : +ve correlation with gastritis • *P. micra* : +ve correlation with periodontal disease • C. rectus: +ve correlation with periodontal disease		• Higher bacterial frequencies observed in the oral cavity after BS • BS has Inverse microbial effect on oral and stomach environments	
Shillitoe et al (2012) 16	2 Weeks after BS: nondiabetics: 2.4-fold increase in the levels of *Bifidobacteria* species Type-2 DM: 10-fold increase in the levels of *Bifidobacteria* species					–		• *Bifidobacteria* : −ve correlation with periodontal disease		• Levels of oral *Bifidobacteria* can reflect that of GIT microbiota • Analysis of oral microbiota can help in providing data with systemic implications	

Abbreviations: +ve, positive; BMI, body mass index; BP, blood pressure; BS, bariatric surgery; DM, diabetes mellitus; F, female; GBS, gastric bypass surgery; GCF, gingival crevicular fluid; M, male; NRR, nothing relevant reported; qPCR, quantitative polymerase chain reaction; RYGB, Roux-en-Y gastric bypass; SG, sleeve gastrectomy; -ve, negative

### Quality Assessment of Included Studies

The quality of included articles was assessed using the quality assessment tools of the National Heart, Lung, and Blood Institute of the National Institutes of Health (NIH) for quality assessment of the Observational Cohort and Cross-Sectional Studies and Controlled Intervention Studies. 31

The criteria for assessment are as follows: Q1., “Was the research question or objective in this paper clearly stated?”; Q2., “Was the study population clearly specified and defined?”; Q3., “Was the participation rate of eligible persons at least 50%?”; Q4., “Were all the patients selected or recruited from the same or similar populations (including the same time period)? Were inclusion and exclusion criteria for being in the study prespecified and applied uniformly to all participants?”; Q5., “Was a sample size justification, power description, or variance and effect estimates provided?”; Q6., “For the analyses in this paper, were the exposure(s) of interest measured prior to the outcome(s) being measured?”; Q7., “Was the timeframe sufficient so that one could reasonably expect to see an association between exposure and outcome if it existed?”; “Q8: For exposures that can vary in amount or level, did the study examine different levels of the exposure as related to the outcome (e.g., categories of exposure, or exposure measured as continuous variable)?”; Q9., “Were the exposure measures (independent variables) clearly defined, valid, reliable, and implemented consistently across all study participants?”; Q10., “Was the exposure(s) assessed more than once over time?”; Q11., “Were the outcome measures (dependent variables) clearly defined, valid, reliable, and implemented consistently across all study participants?”; Q12., “Were the outcome assessors blinded to the exposure status of participants?”; Q13., “Was loss to follow-up after baseline 20% or less?”; and Q14., “Were key potential confounding variables measured and adjusted statistically for their impact on the relationship between exposure(s) and outcome(s)?”

## Results

### Identification and Screening

**Fig. 1:**
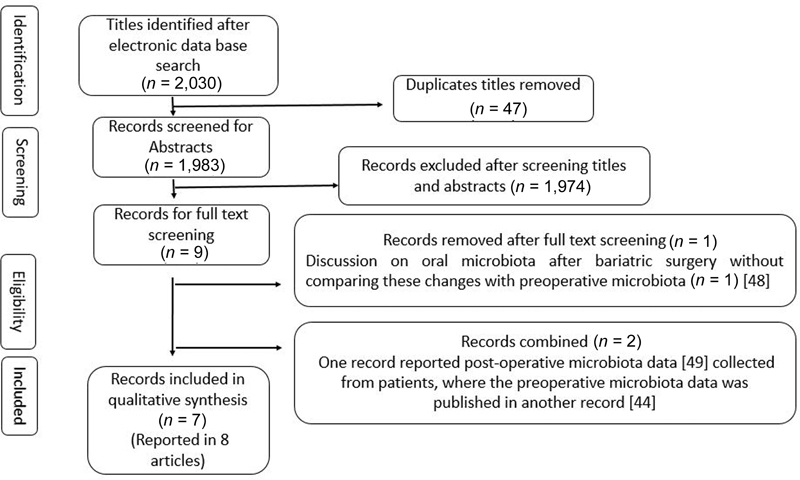
Flowchart of article inclusion strategy based on PRISMA guidelines. PRISMA, the Preferred Reporting Items for Systematic Reviews and Meta-analyses.

The initial electronic database search leads to 2,030 titles ( Table 2 ). A total of 47 titles were found to be duplicated and were removed. Titles and abstracts of 1,983 articles were screened to exclude irrelevant articles (based on inclusion and exclusion criteria). Articles with conflicts were discussed to resolve the disagreements. Kappa score (Cohen's kappa coefficient; k=0.922) indicates a near-perfect agreement between the two reviewers. The full text of the leftover titles was assessed to choose the suitable studies, and, finally, nine articles were shortlisted. A manual search of references for these articles was performed, but no more relevant articles were found. Out of nine selected articles, one was the postoperative microbiota data 32 collected from patients where the preoperative microbiota data were published separately, 28 whereas another study was excluded because it discussed oral microbiota after BS without comparing these changes with preoperative microbiota. 33 Thus finally, seven studies (reported in eight articles) were incorporated into this review. Fig. 1 illustrates the search outcomes.

### Quality Assessment of Included Studies

A total of seven studies were included in this review. The quality of one study was rated as good 13 and six studies were rated as fair 15 16 25 26 27 28 32 with a risk of bias due to the absence of blinding. Results of the NIH quality assessment scale are displayed in Table 4 .

**Table 4:** Quality analysis outcomes of the included studies (NIH quality assessment tool)

Study	Question number													
	1	2	3	4	5	6	7	8	9	10	11	12	13	14
1. Shillitoe et al (2012) 16	Y	Y	N	Y	N	N	Y	NA a	Y	NA	Y	N	Y	NR
2. Hashizume et al (2015) 15	Y	Y	NR	Y	N	Y	Y	NA a	Y	NA	Y	Y	Y	NR
3. Sales-Peres et al (2015) 13	Y	Y	Y	Y	Y	Y	Y	NA a	Y	NA	Y	N	Y	NR
4. Pataro et al (2016) 25	Y	Y	N	Y	Y	N	Y	NA a	Y	NA	Y	N	Y	NR
5. Džunková et al (2020) 26	Y	Y	NR	Y	N	Y	Y	NA a	Y	NA	Y	N	Y	NR
6. Balogh et al (2020) 27	Y	Y	NR	Y	N	Y	Y	NA a	Y	NA	Y	N	Y	NR
7. Stefura et al (2020, 2021) 28 33	Y	Y	NR	Y	N	Y	Y	NA a	Y	NA	Y	N	Y	NR

Abbreviations: N, no; NA, not applicable; NIH, National Institutes of Health; NR, not reported; Y, yes.

a Will not count negatively towards the quality rating.

### Characteristics of Included Studies

All the included studies ( *n* =7) evaluated the effect of BS on oral microbiota in humans. Included studies were published during the last 6 to 7 years (2015–2021; Table 3 ). Three out of seven studies were conducted in Brazil, 13 15 25 and one each was conducted in Poland, 28 32 the Czech Republic, 26 Hungary, 27 and the United States. 16 Sample size researched and varied in these studies from *n* =27 15 to *n* =154. 25 The cumulative number of female participants was higher, and they contributed to 72.9% (290) of the cumulative sample size (398), whereas male participants contributed only 27.1% (108). The mean age of participants ranged from 33.9 27 to 48 26 years, with variation in each study. Four out of seven studies reported the presence of comorbidities in the selected participants (DM, hypertension, and others) 15 16 26 28 33 ; in one study, none of the participants had comorbidities, 26 whereas two studies did not disclose these details. 13 25 With regard to relevant oral findings, two studies reported the presence of periodontitis in sample groups, 13 25 two studies mentioned that there was no periodontitis 16 27 ; in one study, participants were wearing removable dental prosthesis and had dental caries, 15 whereas two studies did not disclose any of these details. 26 28 32

There was a difference in the type of BS used in the selected studies. RYGB 13 15 16 25 was performed in four out of seven studies, in one study, SG was the choice of the surgical technique, 28 four different types of BS procedures were performed in one study on the selected population, 26 whereas one study did not give details about the type of weight loss surgery. 27 The mean BMI of participants in included studies varied from 51.72 15 to 44.99kg/m ^2^ 25 in the pre-BS group/baseline group to 48.4 28 32 to 26.53kg/m ^2^ 25 in post-BS group.

Four out of seven studies compared the change in oral microbiota in the same selected participants before and after BS, 13 15 16 26 whereas three studies 25 27 28 32 compared this change in BS patients with those who have not undergone BS. For qualitative and quantitative analysis of the microbiota, gingival crevicular fluid (GCF) was the source of specimen in two studies, 13 27 stimulated saliva in two, 15 16 unstimulated saliva in one, 26 and oral swabs only were collected in one study. 28 32 One study collected specimens from both unstimulated saliva and the dorsum of the tongue. 25

Out of the total of seven studies, three used the quantitative polymerase chain reaction (qPCR) technique for relative DNA quantification of specific microbial targets, 13 16 25 one expressed microbiological counts as colony-forming units per milliliter (CFU/mL saliva) on selective culture media, 15 one used matrix-assisted laser desorption ionization time of flight mass spectrometry (MALDI-TOF MS) and MALDI Biotyper for identification, 27 whereas two studies used 16S rRNA gene sequence analysis technique. 26 28 32 There are differences in the follow-up between the included studies. Follow-up varied from 1 day 26 to more than 24 months. 25

### Results of the Individual Studies

All seven studies investigated the changes in oral microbiota after BS. These changes were reported as early as 1 day after BS 26 and continued up to 2 years of follow-up. 25 The type of BS was not differentially associated with bacterial diversity or specific changes in the oral microbiota; however, each study reported a marked increase or decrease in certain species after BS. The reported trend of changes in oral microbiota was highly heterogeneous between individuals within each study. Trends in changes in oral microbiota between studies were heterogenous, primarily because only two of the studies 26 28 32 used similar approaches in identifying, and quantitating the oral microbiota. Details of changes in microbiota are described in Table 3 .

*Changes in salivary microbiota* : two studies reported a significant increase in firmicutes (mutans streptococci 15 and *Veillonella atypica* 26 ), one each reported an increase in sac fungi ( *Candida albicans* 15 ), bacteroidetes ( *Porphyromonas gingivalis* and *Tannerella forsythia* 25 ), spirochaetes ( *Treponema denticola* 25 ), and *Bifidobacteria* 16 species. Three studies reported a significant decrease in firmicutes ( *Lactobacillus spp* , 15 *Granulicatella elegans* , 26 and *Parvimonas micra* , 25 one each reported decrease in bacteroidetes species ( *Porphyromonas pasteri* and *Prevotella nanceiensis* ) 26 and proteobacteria ( *Helicobacter pylori* 25 ).

*Changes in GCF microbiota:* Sales-Peres et al 13 reported a significant increase in bacteroidetes species ( *P.* *gingivalis* and *T. forsythia* ) and a significant decrease in spirochaetes ( *T. denticola* ) and bacteroidetes species ( *Prevotella intermedia* ). At the same time, Balogh et al 27 reported a marked increase in firmicutes ( *Streptococcus* ) and sac fungi (albicans and nonalbicans *Candida* ) and a significant decrease in firmicutes ( *Granulicatella* ), actinobacteria ( *Actinomyces* ), and fusobacteria species ( *Fusobacterium* ). Changes in oral scrapings microbiota: increase in bacteroidetes, 25 28 32 proteobacteria, 28 32 actinobacteria, 28 32 and spriocheates 25 was reported in scrapings collected from the oral cavity.

## Discussion

In the current review of literature analyses, the available studies were analyzed to evaluate the changes in oral microbiota in patients after BS and attempted to correlate these alterations in the number and quality of microorganisms, with oral manifestations. To the best of our knowledge, to date, there is no systematic review that assesses the change in oral microbiota after BS. The findings based on the seven selected studies improve our knowledge about the changes in oral microbiota post-BS which may aid in the effective management of changes observed post-BS. The findings support that oral microbiota is altered after BS but this variation varies with the individuals. Thus the hypothesis framed can be rejected.

Oral microbiota consists of various microbial species which colonizes in different areas of the oral cavity. The characteristics of each area determine the configuration of microbiota. 34 There is a critical balance between these microorganisms and the host. In the presence of systemic diseases and /or if oral hygiene is not adequately maintained, this equilibrium gets disturbed, and the quality and quantity of microbiota get altered which may manifest as oral diseases like periodontitis, caries, gingivitis, oral mucosal changes, and others. The bacterial taxa reported to be associated with caries by culture and molecular studies include *Streptococcus* , *Lactobacillus* , *Actinomyces* , phylotypes of *Bifidobacterium* , *Propionibacterium* , and *Atopobium* . 34 35 36 37 38 In contrast, taxa reported to be associated with periodontal disease include *P. gingivalis, T. forsythia, T. denticola, Aggregatibacter actinomycetemcomitans, Fusobacterium nucleatum, Filifactor alocis,* and *P. intermedia* . 34 38 39 40

Obesity is a complex state which involves excessive fat accumulation that can have a negative effect on the overall health of an individual. Vgontzas et al 41 reported that proinflammatory cytokines, which are secreted by fat tissues, are directly proportional to BMI and visceral obesity. This systemic inflammation alters the oral microbiota in obese individuals, which are found to have higher levels of phylum bacteroidetes ( *T. forsythia* and *P. gingivalis)* , 42 43 phylum spirochaetes *(T. denticola)* , 43 phylum firmicutes ( *Granulicatella adiacens* and *Streptococcus oligofermentans* ), phylum actinobacteria (actinomyces), phylum proteobacteria ( *Aggregatibacter* ) as compared with nonobese individuals. 44 In addition to this, comorbidities associated with obesity like type-2 DM, hypertension, hyperlipidemia, and others, also alter the oral microbiota. 16 45 BS is an effective treatment modality to manage morbidly obese patients and their related comorbidities in the long term. 4 Studies have reported a change in oral microbiota 13 15 16 25 26 27 28 32 in patients who have undergone BS procedures. These alterations can be associated with the site of the oral cavity. One of the prerequisites regarding microbiota analysis and comparison between groups is the absence of any relevant disease before intervention, so that observed alteration can be attributed to intervention. 46 In the current review, three studies reported the presence of oral disease preoperatively 13 15 25 and two studies did not disclose any of these details, 26 28 32 Five out of seven studies included antibiotic administration in exclusion criteria. One study did not include it. 26 In another study, 16 the exclusion criteria was those patients who have received antibiotics within the previous 6 months, but during methodology, the authors mentioned administering a single dose of antibiotics to patients. Studies reported that the use of antibiotics can alter the composition of oral microflora 47 48 which can return back to normal after 14 days of antibiotic administration. 49

When changes in salivary microbiota after BS were considered, Hashizume et al 15 reported an increase in *Streptococcus mutans* and *Candida albicans* and a decrease in *Lactobacillus spp* . This Increase in *C. albicans* in their study can be related to the inclusion of patients with comorbidities wearing removable dentures. Pataro et al 25 reported higher oral and lower stomach bacteria frequency in the BS group. They reported a nonsignificant decrease in *H. pylori* and an increase in the frequency of red complex species ( *P. gingivalis, T. forsythia,* and *T. denticola* ) in the bariatric group with a much higher number in patients having periodontitis before BS. Their results were in accordance with Jaiswal et al, 50 who reported no improvement in pocket depth and clinical attachment level after 6 months of BS. Džunková et al 26 reported a significant increase in *V. atypica* and a significant decrease in *P. pasteri* . They concluded that GI microbiota is affected directly by BS, whereas salivary microbiota is altered indirectly. Shillitoe et al 16 reported a 10-fold increase in *Bifidobacteria* species. They reported simultaneous changes in oral and lower GI microbiota which could be due to the correction of the systemic mucosal immune defect after BS and the direct influence of oral microbiota which is continuously swallowed. 51

Concerning changes in GCF microbiota, Sales-Peres et al 13 reported a significant increase in *P. gingivalis* and *T. forsythia* and a significant decrease in *T. denticola* and *P. intermedia* . They reported worsened periodontal conditions 6 months after BS and slight improvement after 12 months of follow-up. Despite reduction in the body's inflammatory response, increased periodontal destruction was related to being due to indirect damage mediated by the immunoinflammatory response. They proposed that these changes could be due to frequent eating, osteoporosis, 52 and nutritional deficiencies which are common after BS. Balogh et al 27 reported a marked increase in germ count of streptococcus, albicans *,* and nonalbicans *Candida* and a significant decrease in *Granulicatella* , *Actinomyces,* and *Fusobacterium.* An increase in the proportion of patients affected by *Prevotella sp* . was also reported. The non-albicans species ( *C. dubliniensis, C. kefyr,* and *C. lusitaniae* ) found were similar to those isolated from the oral cavity of immunosuppressed patients. They concluded that despite changes in oral microbiota after BS, patients are unlikely to develop periodontitis if they have uninflamed periodontal conditions and good oral hygiene maintenance preoperatively.

Concerning changes in oral scrapings microbiota, Stefura et al 28 32 reported more proteobacteria species preoperatively, in the patients who have positive weight loss outcome (% expected weight loss [EWL] >50%), when compared with the patients who have negative weight loss outcome (%EWL<50%), in which actinobacteria species is higher preoperatively. They reported an increase in bacteroidetes, proteobacteria, and actinobacteria species postoperatively. Type of BS and patient's age were important factors in determining the amount of weight loss.

All the included studies had indicated a change in quality and quality of oral microbiota after BS but had dissimilar results when type and number of species were considered. Most of the studies had a common consensus that these changes in oral microbiota are not directly related to BS but could be due to indirect reasons. These reasons could be increased frequency of meals (sucrose), 13 15 26 underreporting of food intake by patient, 33 change in food consistency, 15 change in nutritional composition of food, 33 nutritional deficiencies, 13 altered oral pH due to frequent episodes of gastrooesophageal reflux, 26 use of proton pump inhibitors, 53 change in gut–brain axis regulation, 26 alterations in taste perception, 26 54 presence of systemic diseases/comorbidities/immunological factors, 15 presence of dentures in mouth, 15 oral health status before BS, 27 dental hygiene maintenance, 27 and individual-specific resident bacteria. 26

These changes in oral microbiota can be correlated with oral and general manifestations to some extent. Altered species which have been reported to have a positive correlation with periodontitis include *P. gingivalis, T. forsythia, T. denticola* , *P. intermedia* , phyla bacteroidetes, *Prevotella salivae* , A. actinomycetemcomitans, *P. micra* , and *C. rectus* 25 55 56 57 58 59 60 61 . Whereas *Neisseria* and *Bifidobacteria* have a negative correlation with periodontitis. 62 63 *S. Mutans* , phylum actinobacteria, and *V. atypica* have a positive correlation with dental caries. 64 Whereas, *Megasphaera micronuciformis* and *C. albicans,* to some extent, have a negative correlation with caries. 64 65 *C. albicans* and nonalbicans *Candida* species have a positive correlation with immunosuppression, 66 67 68 *H. pylori* and phyla proteobacteria 67 have a positive correlation with gastritis, and *P. gingivalis* has been positively related to cardiovascular diseases. 66 67

Knowledge of changes in oral microbiota and their relation to GI microbiota is very important. The oral cavity can act as an extra gastric pool for many microorganisms. These oral microorganisms can influence GI microbiota and other vital organs of the body directly or indirectly, causing various systemic complications. 69 70 71 72 73 74 Studies have reported three pathways for oral–gut allocation of microbiota 75 76 as follows: (1) direct invasion of the intestinal tract through the esophagus by oral microbiota; (2) through the blood cycling route, pathogenic oral microorganisms, which cause periodontitis, can enter the systemic circulation through the periodontal blood and may act on the whole body, and (3) low-grade inflammatory state caused by the metabolites of oral microbiota that enter the bloodstream and the systemic circulation. Also, it is easier/convenient to obtain oral specimens as compared with faecal specimens in long-term follow-up cases to evaluate the changes in the microbiota.

A dentist can play a vital role in monitoring the oral cavity of patients before and during follow-up visits after BS. It is evitable that at all stages, good oral health should be maintained for these patients to improve their chewing efficiency to keep pathogenic species under control and to reduce systemic complications due to bacteremia. Further long-term studies focusing on monitoring oral microfloral changes and identifying optimal oral microfloral composition after BS may help in better management of these patients.

The outcomes from the current study are also dependent on the different duration of follow-up and different approaches used by the selected articles. The follow-up period varied from 1 day 26 to 2 years, 25 and the location of oral specimen collection was also varied. There was a large variation in sample size and most of the studies had a higher number of female patients. 15 16 25 26 27 28 32 Thus generalization of outcomes was difficult. Also, there was no consistency in the study groups. Due to these limitations, meta-analysis was not feasible. The detailed study selection approach followed is the key point of this review. All studies related to BS and changes in oral microbiota were analyzed, thus making sure that no relevant study is missed.

## Conclusion

The outcomes of this systematic review indicate that considerable changes take place in oral microbiota after BS which can be correlated with common oral manifestations. These changes are mainly due to the indirect effect of BS and may vary with the individuals. Due to variations in the included studies, it is difficult to proclaim any persistent pattern of oral microbiota found after BS. Further long-term investigations are required to get a better picture of the altered microbiota.
